# Tap water perceptions and water filter use vary with socio-demographic characteristics and are associated with water and sugar-sweetened beverage consumption in university students

**DOI:** 10.1017/S1368980023001659

**Published:** 2023-11

**Authors:** Melissa J Slotnick, Julia A Wolfson, Cindy W Leung

**Affiliations:** 1 Department of Nutritional Sciences, The University of Michigan School of Public Health, Ann Arbor, MI 48109, USA; 2 Department of International Health, Johns Hopkins Bloomberg School of Public Health, Baltimore, MD, USA; 3 Department of Health Policy and Management, Johns Hopkins Bloomberg School of Public Health, Baltimore, MD, USA; 4 Department of Nutrition, Harvard T.H. Chan School of Public Health, Boston, MA, USA

**Keywords:** Bottled water, SSBs, Tap water, University students, Water filter, Water insecurity

## Abstract

**Objective:**

The goal of this study is to evaluate university students’ perceptions of tap water safety and water filter use and determine how these perceptions and behaviours affect water and sugar-sweetened beverage intake.

**Design:**

Cross-sectional; online survey conducted in Fall 2021.

**Setting:**

A large, public Midwestern university in the USA.

**Participants:**

Seven-hundred ninety-three university students.

**Results:**

Students who experienced food insecurity, were on a Pell grant, were first-generation college students or were racial/ethnic minorities were less likely to trust tap water safety. Tap water filtration behaviour also varied by age and race/ethnicity. Students who did not agree with the statement ‘my local tap water is safe to drink’ had lower odds of consuming ≥ 3 cups of total water per day (OR = 0·45, 95 % CI: 0·32, 0·62), lower odds of consuming tap water ≥ 3 times/d (OR = 0·46, 95 % CI: 0·34, 0·64), higher odds of drinking bottled water ≥ 1 time per day (OR = 1·80, 95 % CI: 1·22, 2·66) and higher odds of drinking SSB ≥ 1 time per day (OR = 1·47, 95 % CI: 1·01, 2·14) than those who agreed. Students who always or sometimes filtered their tap water had lower odds of consuming ≥ 3 cups of total water per day (OR = 0·59, 95 % CI: 0·39, 0·90) than students who never filtered their tap water.

**Conclusions:**

Tap water perceptions and behaviours affect tap and bottled water and SSB intake among university students. Tap water perceptions and behaviours in this demographic provide important context for university programming promoting healthy beverage initiatives.

Approximately 20 % of the population in the USA does not drink tap water regularly^(1)^. Lower tap water consumption in the US is associated with lower income and education levels, identifying as Black, Asian or Hispanic races, and being born outside of the US^(1,2)^. Distrust in water safety in these populations^(3,4)^ likely contributes to these notable differences in water consumption behaviour^(1)^.

Disparities in sugar-sweetened beverage (SSB) intake, a major source of sugar intake for young adults^(5)^, mimic disparities in tap water consumption. SSB intake is higher in young adults from minority racial/ethnic groups, lower-education and lower-income populations^(5)^. Food insecurity among university students has also been associated with higher SSB consumption^(6)^ and added sugar consumption from SSBs^(7)^. Higher water intake has been associated with lower SSB intake; however, these benefits are not observed across all socio-economic subgroups^(8)^.

Water filtration could be a strategy to increase tap water consumption. Water filter use at home has been associated with higher odds of drinking tap water and lower odds of SSB consumption in US adults^(9,10)^. However, information on water filter use in the USA is limited^(10)^, and no known studies have investigated how attitudes towards water safety and water filtration use affect beverage intake among young adults or in university settings.

The university environment is a unique setting for studying dietary behaviours and perceptions among young adults. Students are often living away from home for the first time and independently making food and beverage choices. Such choices have potential to influence their future dietary patterns and purchasing decisions^(11–13)^.

The current study has two objectives: (1) to determine how perceptions of tap water safety and water filter use vary with socio-demographic variables in university students and (2) to evaluate how perceptions affect water and SSB intake. It is hypothesised that distrust of tap water safety will be associated with lower water and higher SSB intake, and water filter use will be associated with higher water and lower SSB intake. Understanding how perceptions, behaviours and disparities affect beverage consumption in this population can help to shape healthy beverage initiatives in secondary institutions and lend understanding to health disparities.

## Methods

### Study participants

In Fall 2021, an online (Qualtrics) survey was fielded to students recruited from the university registrar’s office, oversampling first-generation and racial/ethnic minority students. The study purpose was ‘[to] improve our understanding of students’ demographics and health behaviours.’ Totally, 3782 students were contacted and 885 students completed the survey, yielding a response rate of 23 %. Students received a $10 Amazon gift card for survey completion. Those with incomplete responses were excluded from analysis, leaving an analytic sample of 793 participants.

### Measures

#### Tap water perceptions and filter use

Tap water perceptions were assessed based on questions modified from the 2010 ConsumerStyles and Youth-Styles mail surveys^(4)^. Students were asked the question ‘my local tap water is safe to drink.’ For analysis, response options were grouped into two categories: agree/strongly agree and ‘do not agree’ (strongly disagree/disagree/neutral). Water filter use was assessed with the following question: ‘I filter my tap water before drinking it.’ For analysis, response choices of ‘always’ and ‘sometimes’ were grouped together, and ‘never’ was coded as ‘no.’

#### Socio-demographic covariates

Students self-reported age, receipt of Pell grant in the current academic year (yes/no), gender (man, woman, other/non-binary) and race/ethnicity (Asian/PI, Black, Hispanic, Other/Multiracial and White/MENA). MENA was grouped with White according to US Census guidelines^(14)^. Food security was assessed using the ten-item U.S. Adult Food Security Survey Module, and low and very low food security were collectively termed food insecurity^(15)^. Federal Pell grants are usually awarded to students with exceptional financial need and are therefore an indicator of income level.

#### Beverage intake

Beverage consumption was assessed for fifteen types of beverages using the validated BEVQ-15^(16)^, modified as previously described^(7)^. Beverages assessed included SSB (regular soda, energy/sports drinks, fruit-flavoured drinks, sweetened tea, sweetened coffee and flavoured milk) and other beverages (diet soda/pop/fruit drinks/energy drinks/sport drinks, 100 % pure fruit juice, plain/unflavoured milk, artificially sweetened or unsweetened tea and coffee, plain bottled water, plain tap water and plain/flavoured unsweetened sparkling water). Students self-reported intake frequencies ranging from ‘never’ to ‘6 or more times per day,’ and the usual volume consumed each time ranging from ‘less than 6 fl. oz. (0·75 cup)’ to ‘more than 20 fl. oz. (2·5 cups)’ for beverages and total water (plain tap water, bottled water and sparkling water). Ounces consumed per day were calculated for each beverage and total water. SSB intake (oz./d) was calculated by summing intake of regular soda, energy/sports drinks, fruit-flavoured drinks, sweetened tea, sweetened coffee and flavored milk. Ounces were converted to l/d for presentation of results. For the BEVQ-15, volume consumed is not recorded for tap water and bottled water, so frequency of intake (times/d) was used for analysis of tap and bottled water. For logistic regression analysis, to compare with previous research^(10)^ and to allow for separation of regular consumers and non-consumers due to variable distribution in the current study, beverage consumption was classified into the following categories: total water intake ≥ 3 cups (710 ml)/d, SSB intake ≥ 1 serving (12 oz. or 355 ml)/d, bottled water intake ≥ 1 time/d and tap water intake ≥ 3 times/d.

### Statistical analysis

*χ* ^2^ tests were used to evaluate differences in water perceptions and behaviours by socio-demographic characteristics. Differences in mean beverage intake for each water perception/behaviour were calculated using two-sided *t* tests. Associations between water perceptions and behaviours and beverage intake were assessed using multivariate logistic regression. Models were adjusted for age, gender, race/ethnicity, first-generation status, food insecurity status and Pell grant status. Interactions between socio-demographic variables and total water and SSB intake were evaluated. Missing values (*n* 17) were assigned an indicator value. All statistical tests were two-sided, performed using SAS, version 9.4^(17)^ and significance was considered at *P* < 0·05.

## Results

Younger students were less likely to agree that their tap water was safe to drink than older students (*P* < 0·001), and Asian/PI, Black and Hispanic students were less likely to agree that their tap water was safe to drink than White/MENA students (*P* < 0·001) (Table 1). A higher percentage of students who did not agree their tap water was safe to drink were first generation (57 %) *v*. not first generation (41 %) (*P* < 0·001) and on a Pell grant (56 %) *v*. not on a Pell grant (44 %) (*P* = 0·001). Food-insecure students were less likely to agree that their tap water is safe to drink than food secure students (*P* < 0·001).

**Table 1:** Socio-demographic characteristics of undergraduate students at a large, public Midwestern university and mean beverage intake, stratified by drinking water safety perception and water filter use^*^

				Tap water is safe to drink					Filter tap water				
		Total n (%)		Agree		Do not agree^†^			Never		Always/Sometimes		
		*n*	%	*n*	%	*n*	%	*P* value^‡^	*n*	%	*n*	%	*P*-value
Total (%)		793		502		291			171		622		
Age													
≤ 18 years		98	12·3	50	10·0	48	16·5	< 0·001	19	11·1	79	12·7	0·025
19–20 years		171	21·6	83	16·5	88	30·2		25	14·6	146	23·5	
≥ 21 years		524	66·1	369	73·5	155	53·3		127	74·2	397	63·8	
Gender													
Man		287	36·2	195	38·8	92	31·6	0·102	65	38·0	222	35·7	0·746
Other/Non-Binary		24	3·0	16	3·2	8	2·7		4	2·3	20	3·2	
Woman		482	60·8	291	58·0	191	65·6		102	59·7	380	61·2	
Race/ethnicity													
Asian/PI		309	38·9	179	35·7	130	44·7	< 0·001	39	22·8	270	43·4	< 0·001
Black		56	7·0	32	6·4	24	8·2		23	13·5	33	5·3	
Hispanic		82	10·3	32	6·4	50	17·2		16	9·4	66	10·6	
Other/Multi-racial^§^		59	7·4	41	8·2	18	6·2		11	6·4	48	7·7	
White or MENA^‖^		287	36·2	218	43·4	69	23·7		82	48·0	205	33·0	
First generation^¶^													
No		435	56·1	315	62·7	120	41·2	< 0·001	101	60·5	334	54·8	0·194
Yes		341	43·9	176	35·1	165	56·7		66	39·5	275	45·2	
Pell grant^**^													
No		420	53·0	292	58·2	128	44·0	< 0·001	96	56·1	324	52·1	0·347
Yes		373	47·0	210	41·8	163	56·0		75	43·9	298	47·9	
Food insecure													
No		631	79·6	428	85·3	203	69·8	< 0·001	134	78·4	497	79·9	0·658
Yes		162	20·4	74	14·7	88	30·2		37	21·6	125	20·1	
		Total Mean	sd	Mean	sd	Mean	sd	*P* value	Mean	sd	Mean	sd	*P*-value
												
Total water intake	(oz/d)	50·0	43·8	54·5	40·1	42·5	48·9	< 0·001^††^	48·1	44·3	57·4	41·6	0·013^‡‡^
	(l/d)	1·48	1·30	1·61	1·19	1·26	1·44		1·42	1·31	1·70	1·23	
SSBs intake	(oz/d)	9·2	17·9	7·7	13·2	12·2	23·9	0·003^††^	9·5	19·1	8·7	13·2	0·531^††^
	(l/d)	0·27	0·53	0·23	0·39	0·36	0·71		0·28	0·56	0·26	0·39	
		Total Mean	sd	Mean	sd	Mean	sd	*P* value	Mean	sd	Mean	sd	*P*-value
Bottled water intake (times/d)		0·7	1·5	0·5	1·2	1·0	1·7	< 0·001^††^	0·7	1·4	0·8	1·5	0·501^‡‡^
Tap water intake (times/d)		3·3	2·3	3·7	2·2	2·5	2·4	< 0·001^‡‡^	3·6	2·3	3·2	2·3	0·031^‡‡^

* Column percentages represent percent of students falling into drinking water perception category or filter use category, total percentages represent percent of total sample population. Missing data were removed from analyses.

† Includes those who neither agree nor disagree with the statement ‘My local tap water is safe to drink’.

‡ *P* value indicates statistical significance of comparisons between groups using X^2^ tests.

§ Includes students reporting race/ethnicity as Other (self-defined) or Multiracial/Multiethnic.

‖ Middle Eastern/North African.

¶ Students were classified as first-generation college students if the highest level of education of both parents was high school/GED. A missing data indicator was used for *n* 17 participants missing data on first-generation status.

** Students receiving a Federal Pell grant; Federal Pell grants are usually awarded to students with exceptional financial need and are therefore an indicator of lower income level.

†† Two-sample *t* test for equality of means, unequal variances.

‡‡ Two-sample *t* test for equality of means, equal variances.

Tap water filtration behaviour also varied by age and race/ethnicity. Students < 21 years old were more likely to filter their tap water than those ≥ 21 (*P* = 0·03). A higher percentage of Asian students filtered their water (43 %) than did not (23 %), but a higher percentage of Black students did not filter their water (13·5 %) than filtered their water (5·3 %) (*P* < 0·001).

Mean total water intake was higher for students who agreed their tap water was safe (mean = 54·5 ± 40·1 oz./d or 1·61 ± 1·19 l/d), *v*. those who did not (mean = 42·5 ± 48·9 oz./d or 1·26 ± 1·44 l/d) (*P* < 0·001). Frequency of tap water intake demonstrated a similar pattern. Mean SSB intake was lower for students who agreed that their tap water was safe to drink (mean = 7·7 ± 13·2 oz./d or 0·23 ± 0·39 l/d) than for those who did not (mean = 12·2 ± 23·9 oz./d or 0·36 ± 0·71 l/d) (*P* = 0·003). Mean frequency of bottled water intake was also lower for students who agreed that their water was safe to drink (mean = 0·5 times/d, sd = 1·2) compared with those who did not (mean = 1·0 times/d, sd = 1·7) (*P* < 0·001).

Students who filtered their tap water had higher mean total water intake (mean = 57·4 ± 41·6 oz./d or 1·70 ± 1·23 l/d) than students who did not (mean = 48·1 ± 44·3 oz./d or 0·26 ± 0·39 l/d) (*P* = 0·01). Similarly, students who filtered their tap water had a higher mean frequency of tap water intake (mean = 4·4 times/d, sd = 2·1) than students who did not (mean 4·0 times/d, sd = 2·5) (*P* = 0·03). Mean SSB intake and mean frequency of bottled water intake did not differ by tap water filtration.

Students who did not agree with the statement ‘my local tap water is safe to drink’ had lower odds of consuming ≥ 3 cups of total water per day (OR = 0·45, 95 % CI: 0·32, 0·62), lower odds of consuming tap water ≥ 3 times/d (OR = 0·46, 95 % CI: 0·34, 0·64), higher odds of drinking ≥ 1 SSB serving per day (OR = 1·47, 95 % CI: 1·01, 2·14) and higher odds of drinking bottled water ≥ 1 time per day (OR = 1·80, 95 % CI: 1·22, 2·66) than those who agreed (Table 2). Students who filtered their tap water had lower odds of consuming ≥ 3 cups of total water per day (OR = 0·59, 95 % CI: 0·39, 0·90) than students who did not. Tests for interactions between socio-demographic variables and total water intake were NS (*P*s > 0·05).

**Table 2:** Adjusted odds ratios (OR) with 95 % CI and mean intake data for drinking water behaviours and beverage consumption among undergraduate students at a large, public Midwestern university (*n* 793)^*^

Beverage Consumption	Tap water is safe to drink				Filter tap water			
	Agree (*n* 502)		Do not agree (*n* 291)		No (*n* 171)		Always/Sometimes (*n* 622)	
	OR	95 % CI	OR	95 % CI	OR	95 % CI	OR	95 % CI
Total water intake ≥ 3 cups/d	Ref.	–	0·45	0·32, 0·62	Ref.	–	0·59	0·39, 0·90
SSB intake ≥ 1 serving/d^†^	Ref.	–	1·47	1·01, 2·14	Ref.	–	1·11	0·71, 1·73
Bottled water intake ≥ 1 time/d	Ref.	–	1·80	1·22, 2·66	Ref.	–	0·70	0·44, 1·10
Tap water intake ≥ 3 times/d	Ref.	–	0·46	0·34, 0·64	Ref.	–	0·79	0·55, 1·13

* All models were adjusted for age, gender, race/ethnicity, first-generation status, Pell grant status and food insecurity status. *n* 17 respondents were missing data on first-generation status and were retained in the model using a missing value indicator.

† Serving defined as twelve fluid ounces (355 ml).

## Discussion

Students who were food insecure, on a Pell grant, first generation or racial/ethnic minorities were less likely to trust tap water safety. In the USA, higher tap water mistrust has been observed among people who are Hispanic, non-Hispanic Black, lower income and immigrants^(3,18,19)^. Furthermore, tap water avoidance has been associated with food insecurity in the USA^(20)^. Our results confirm that similar attitudes persist in this university student/young adult population; however, it should be noted that due to sampling methods, this sample had a higher percentage of Asian (38·9 *v*. 19 %), Black (7·0 *v*. 4 %), Hispanic (10·3 *v*. 8 %), Pell grant recipients (47·0 *v*. 19 %) and first-generation students (43·9 *v*. 16 %) than the general student body.

In a previous study, SSB warning labels on the current university campus led to a decrease in SSB intake, but no change in water intake^(7)^. We observe that tap water distrust is associated with lower total water and tap water intake, and thus plays a role in student beverage choices. Students distrusting tap water had higher mean SSB intake, and tap water distrust was associated with higher SSB intake in the multivariate adjusted model.

We hypothesised that tap water filtration could be a strategy to increase tap water intake, decrease bottled water intake and/or decrease SSB intake. Previously, US adults using a water filter had a higher odds of drinking tap water and lower odds of consuming SSB^(10)^, and filter use was associated with increased water and decreased SSB intake in a recent intervention^(21)^, yet our results do not corroborate the findings. It is possible that filtering tap water does not negate safety concerns in this population. Given a large percentage of the study population drinks filtered water (78 %), other factors – such as access, palatability or beverage availability – may be influencing results. Further research is necessary to understand beverage choice behaviours in this population.

Colleges present an opportune setting to improve beverage consumption habits in young adults, and some university initiatives to improve water access, reduce SSB intake, and promote sustainability have proven effective^(22–24)^. To improve campus sustainability, bottled water reduction strategies have been implemented at several universities^(25–29)^ yet do not always result in increased tap water intake^(26,27)^. The current study emphasises the need to consider how socio-demographic disparities in tap water safety perceptions affect consumption and to account for such disparities in healthy beverage initiatives. For example, future studies focusing on water quality messaging, access and perceptions driving beverage choices have potential to improve effectiveness of current initiatives.

Strengths of this study include large sample size and refined beverage intake measurements. This study has several limitations. Amounts consumed per serving were not available for tap and bottled water, resulting in different intake measures for different water sources. Tap water quality data were also unavailable. The survey did not distinguish between types of water filters or assess awareness of campus water filters. This study is limited to university students and may not be representative of all young adults. Lastly, as students in Michigan, attitudes in this population may be disproportionately affected by the Flint Water Crisis^(30)^.

This study lends important insight into how water-quality perceptions and behaviours affect tap water, bottled water and SSB intake in young adults and university students. The findings provide a launching point for university programming promoting healthy beverage initiatives and drinking water access in diverse student populations.
